# Screening protein – Single stranded RNA complexes by NMR spectroscopy for structure determination^☆^

**DOI:** 10.1016/j.ymeth.2013.09.018

**Published:** 2014-02

**Authors:** Jaelle N. Foot, Mikael Feracci, Cyril Dominguez

**Affiliations:** Department of Biochemistry, Henry Wellcome Laboratories of Structural Biology, University of Leicester, UK

**Keywords:** NMR spectroscopy, Protein–RNA complex, STAR proteins, Sam68, T-STAR

## Abstract

In the past few years, RNA molecules have been revealed to be at the center of numerous biological processes. Long considered as passive molecules transferring genetic information from DNA to proteins, it is now well established that RNA molecules play important regulatory roles. Associated with that, the number of identified RNA binding proteins (RBPs) has increased considerably and mutations in RNA molecules or RBP have been shown to cause various diseases, such as cancers. It is therefore crucial to understand at the molecular level how these proteins specifically recognise their RNA targets in order to design new generation drug therapies targeting protein–RNA complexes. Nuclear magnetic resonance (NMR) is a particularly well-suited technique to study such protein–RNA complexes at the atomic level and can provide valuable information for new drug discovery programs. In this article, we describe the NMR strategy that we and other laboratories use for screening optimal conditions necessary for structural studies of protein-single stranded RNA complexes, using two proteins, Sam68 and T-STAR, as examples.

## 1 Introduction

While RNA molecules have long been considered as passive molecules that transfer information from genes to proteins, the last few years have seen the emergence of a massive but still poorly understood RNA world. For example, recent studies from the ENCODE project (http://encodeproject.org) suggested that, while only 1.5% of our genome corresponds to protein-coding sequences, between 20% and 80% of it is transcribed into RNA [1]. It is now clear that RNA molecules are highly abundant and play crucial roles in multiple cellular functions [2], [3]. Associated with this, the number of RNA binding proteins (RBPs) identified has also increased significantly in the last decade. Recently, more than 800 human proteins have been identified that directly bind messenger RNAs (mRNAs) [4], [5]. While half of these proteins contain well-known RNA binding domains (RBDs) such as the RNA recognition motif (RRM), the double-stranded RNA binding domain (dsRBD) or the hnRNP K homology (KH) domain, the other half were not previously predicted to be RNA binding proteins. Mutations found in RNA and RBPs have been shown to cause numerous diseases such as neurological disorders, genetic diseases and cancers [6], [7], [8]. It is therefore crucial to obtain structural information of these protein–RNA complexes in order to, first understand the specificity of recognition, and second to target these complexes for novel therapeutic strategies.

**Fig. 1:**
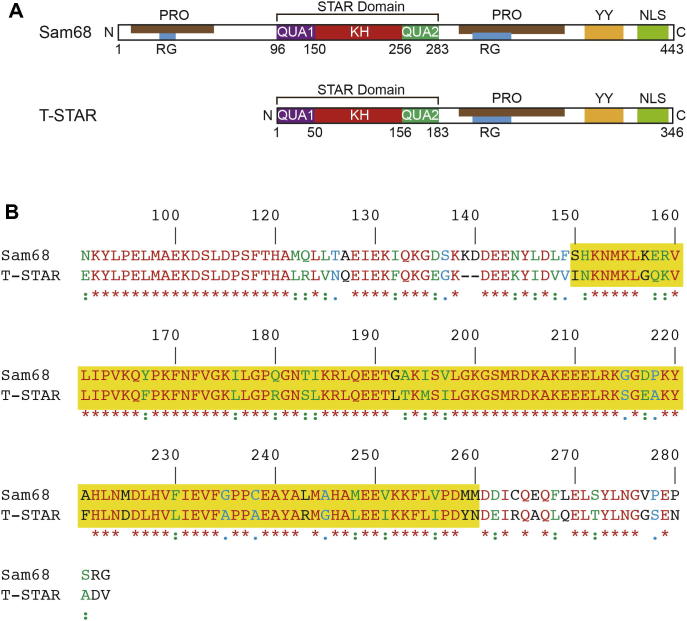
Domain organisation of Sam68 and T-STAR. (A) Sam68 and T-STAR contain a STAR domain responsible for RNA binding that is composed of a central KH domain flanked by QUA1 and QUA2 regions. In addition, these proteins contain various motifs necessary for the cellular function of the protein. The nuclear localisation signal (NLS) allows the proteins to shuttle between the cytoplasm and the nucleus. The proline-rich (PRO) and tyrosine-rich regions (YY) are necessary for tyrosine phosphorylation of these proteins and the arginine–glycine rich regions (RG) are target sites for arginine methylation. (B) Sequence alignment of Sam68 and T-STAR STAR domains. The amino acids of these two proteins in this region are 69% identical (red), 16% highly homologous (green) and 5% homologous (blue). The alignment was performed using CLUSTALW. The KH domain is highlighted in yellow.

RNA molecules are single stranded in cells and a majority of RBPs recognise and bind short single-stranded RNA (ssRNA) motifs through specific contacts with the nucleic acid bases. The two major techniques to solve structures of macromolecular complexes such as protein-ssRNA complexes are X-ray crystallography and nuclear magnetic resonance (NMR). While overall, NMR contributes to around 10% of the structures deposited into the protein data bank (PDB), the contribution of NMR for structure determination of protein-ssRNA complexes is 53% (out of 62 protein-ssRNA complexes, 33 were solved by NMR and 29 by X-ray crystallography). This emphasises that NMR is a major technique for the investigation of such complexes. This fact can be explained by numerous intrinsic properties of protein-ssRNA complexes [9], [10], [11]. First, most known RBPs contain small globular RBDs, such as the RRM or the KH domains, that are around 100 amino acids in length and therefore suitable for NMR studies. Second, single-stranded RNA molecules are highly flexible which can interfere with the crystallisation process of such complexes. Third, many RBPs are modular proteins containing more than one RBD separated by flexible linkers. The presence of such flexible regions and the lack of well-defined relative orientation of the RBDs can also prevent formation of crystals. Additionally, while full-length RBPs often bind RNA with high affinity, they act through a modular interaction approach where each RBD binds rather weakly to its RNA substrate (sometimes in the millimolar range) and the high affinity is provided by the presence of multiple RBDs within one RBP. Therefore RBD-ssRNA complexes are often dynamic and can prevent the formation of a well-defined crystal of the complex, while still being suitable for NMR studies. Finally, although new methodological developments have allowed a precise definition of the RNA sequence specifically recognised by RBPs or RBDs [12], these sequences are often degenerate and the identification of the optimal ssRNA sequence for structural studies is far from being straightforward. From that point of view NMR is very powerful because it allows the screening of multiple RNA sequences at an early stage of the structural investigation, as will be detailed in this article for two proteins, Sam68 and T-STAR, that belong to the STAR family of proteins [13], [14]. Sam68 is the best-characterised member of this family and is involved in various post-transcriptional regulation events, such as alternative splicing and RNA export [15], [16]. T-STAR, also known as SLM2, is closely related to Sam68 but its biological function is less well characterised [17], [18]. T-STAR was recently identified as a specific neuronal splicing factor [19]. STAR proteins are characterised by the presence of a STAR domain necessary for RNA binding [13]. This domain can be subdivided into a central KH domain flanked by highly conserved regions, QUA1 and QUA2 (Fig. 1). Previous structural studies on other STAR proteins indicated that the KH-QUA2 region of the STAR domain is involved in RNA binding and the QUA1 region is involved in the homo-dimerisation of the protein [20], [21], [22], [23], [24]. While KH domains generally accommodate 4 nucleotides [25], the NMR structure of SF1 KH-QUA2 demonstrated that the QUA2 region adopts an α-helical conformation packed against the KH domain and interacts with three additional nucleotides [20]. This large RNA interaction involving the QUA2 region was recently confirmed by the X-ray structures of GLD-1 and Quaking STAR domains in complex with RNA [22]. In that case, the KH-QUA2 region accommodates five and six nucleotides, respectively. While SF1, Quaking and GLD-1 specifically bind similar RNA sequences containing a CUAAC motif, SELEX data indicate that Sam68 and T-STAR specifically recognise A/U rich RNA sequences [26], [27]. Consistently, sequence alignment between STAR proteins suggested that the RNA binding mode of Sam68 and T-STAR might be different to other STAR proteins [28]. Additionally, because the QUA2 amino acids of Quaking and GLD-1 that interact with the RNA are not conserved in Sam68 and T-STAR, it has been proposed that the QUA2 region of Sam68 might not be involved in RNA binding [28]. This is supported by previous data showing that a construct of Sam68, QUA1-KH, lacking the QUA2 region is able to bind RNA as well as the full-length protein [26] and SELEX data indicating that Sam68 specifically binds four nucleotides as opposed to the six nucleotides bound by GLD-1, and QKI [27].

In this article, we will describe the strategy that we and other NMR laboratories commonly use to define optimal protein constructs and RNA sequences for structural studies, using Sam68 and T-STAR proteins as examples.

## 2 Materials and methods

### 2.1 RNA production

The RNA oligonucleotides used for NMR studies were chemically synthesised at a 1 micromole scale (Dharmacon, Thermo Scientific), deprotected according to manufacturer instructions, and lyophilised. RNAs were then resuspended in 100 μl of water and pH was adjusted to 6.5 or 7.0. RNA concentration was measured by OD_260_ using the theoretical extinction coefficient provided by Dharmacon. Typical RNA stock concentrations ranged between 1 and 4 mM.

### 2.2 Protein production

Sam68 STAR (amino acids 97-283), KH-QUA2 (150-283) and KH (150-260) domains and T-STAR STAR (1-183), KH-QUA2 (50-183) and KH (50-160) domains (Fig. 1) were cloned by the University of Leicester Protein Expression Facility (PROTEX, http://www2.le.ac.uk/departments/biochemistry/research-groups/protex) using the pLeics03 expression vector that contains an N-terminal poly-histidine tag followed by a tobacco-etch virus protease (TEV) cleavage site. All plasmid constructs were verified by DNA sequencing (PNACL, Leicester). Recombinant plasmids were transformed into Rosetta BL21 DE3 cells and expressed in 4 litres of 2TY medium or M9 minimal medium supplemented with ^15^NH_4_Cl. At an optical density of 0.5, cultures were transferred to an incubator at 20 °C for 1 h and protein expression was induced with 400 μM IPTG for 16 h at 20 °C.

The proteins of interest were purified by affinity chromatography using Ni-NTA agarose (Qiagen) followed by TEV cleavage during overnight dialysis in phosphate buffer (20 mM sodium phosphate pH7, 100 mM sodium chloride, 10 mM β-mercaptoethanol) at 4 °C. Because short ssRNA oligonucleotides are easily prone to degradation, 5 μl SUPERase IN RNase Inhibitor (Invitrogen) was added to the protein sample that was further purified by size-exclusion chromatography on a Superdex 75 10/300 (GE Healthcare) into the desired buffer for NMR analysis (see Section 3). Selected fractions were pooled and concentrated (Millipore 10 kDa centricon) to approximately 0.2 mM for NMR studies or to approximately 10 mg ml^−^^1^ for crystallisation screenings. Protein concentrations were estimated by measuring the OD_280_ and using a theoretical extinction coefficient (web.expasy.org/protparam/) derived from the protein sequence. RNAse activity was evaluated using Ambion RNAseAlert Lab Test kit according to manufacturer instructions. It is important to note that RNAse inhibitors should not be added to the final NMR sample because the storage buffer contains components with non-labile protons that interfere with the NMR measurements of the proteins and RNAs.

### 2.3 NMR measurements

NMR samples consisted of 330 μl of proteins at concentrations of at least 200 μM in different buffers and 20 μl of D_2_O. NMR measurements were performed using Bruker AVIII-500 MHz, AVIII-600 MHz, AVIII-600 MHz (equipped with a cryoprobe) and Avance-800 MHz (equipped with a cryoprobe) spectrometers. Data were processed using XWINNMR (Bruker) and analyzed with Sparky (http://www.cgl.ucsf.edu/home/sparky/).

Optimisation of the buffer and temperature conditions as well as the protein constructs and RNA sequences were evaluated using 2D ^15^N–^1^H HSQC experiments for visualizing the ^15^N-labeled protein signals, and 2D-TOCSY and 2D-NOESY experiments to visualise the RNA signals and the presence of intermolecular NOEs. TOCSY and NOESY experiments were recorded in D_2_O with mixing times of 50 and 150 ms, respectively.

### 2.4 X-ray crystallography

Six different crystallisation screens (Proplex, NR-LBD, Morpheus, PACT, JCSG+ and Stura & Macrosol) have been used with different protein and RNA concentrations (10 and 20 mg mL^−^^1^) using the Douglas Instrument Oryx 4 robot. For optimisation, T-STAR KH crystals were grown by sitting drop vapour diffusion at 4 °C in 200 mM ammonium sulfate, 100 mM HEPES pH 7.5 and 20% PEG 3350. Crystals were flash-frozen in mother liquor containing 15% MPD as a cryoprotectant. The KH–AAAUAA complex was crystallised in 2 M lithium sulfate and 100 mM Tris pH7.0 at 4 °C using the sitting drop vapour diffusion at a protein concentration of 15 mg mL^−^^1^ (protein:RNA molar ratio of 1:1.5). Data were collected on single crystals at the diamond synchrotron beamline I04 and microfocus beamline I24 and processed using XDS [29].

## 3 Results and discussion

### 3.1 Defining the optimal protein construct for NMR studies

NMR spectroscopy is limited by the size of the system under study. The upper molecular weight limit for structure determination is currently approximately 50 kDa, which means that it is often not possible to study a full-length protein by NMR. Fortunately, most RBPs are composed of small structurally independent domains that are sufficient for RNA binding. It is therefore possible to subclone RBPs into distinct domains whose size is suitable for NMR studies. If little is known about this RBP, potential functional domains can be identified using multiple sequence alignment algorithms, secondary structure prediction and identification of conserved domains. For well-characterised RBDs, specific constructs can easily be designed. Different protein constructs, although highly homologous, can however behave differently and various expression and purification strategies may have to be attempted in order to obtain a highly concentrated, pure and soluble sample suitable for NMR studies. If protein yield is too low following overexpression in a bacterial host, it may be that the construct is toxic to the cells or prone to aggregation. In this case, adjustment of the domain boundaries, use of an alternative affinity tag or use of a solubility tag may result in a more stable sample [30], [31]. Other options for protein production such as baculovirus or mammalian cells are still not commonly used for NMR studies because isotope labelling is either not possible or not financially viable [32]. The ^15^N–^1^H HSQC is the most commonly used NMR experiment to investigate the suitability of a protein construct for further NMR studies and for investigating the complex formation between a protein and its partner, such as protein, RNA, DNA or small molecules. Such experiments requires a ^15^N-labeled protein that can be obtained by expressing the protein in *Escherichia coli* grown in a minimum medium in which the sole nitrogen source is provided by the addition of ^15^NH_4_Cl. The ^15^N–^1^H HSQC is a two-dimensional NMR experiment that allows a magnetisation transfer between a proton and its attached NMR visible ^15^N isotope. This results in a spectrum in which each NH and NH2 groups give a crosspeak at the specific frequency of the proton in one dimension and the nitrogen in the other dimension. These frequencies are dependent on the atom chemical environments and therefore, in folded proteins, different atoms have different frequencies. Since every amino acid (except proline and the N-terminal amino acid) contains an amide group in its backbone, this spectrum is often referred as the NMR fingerprint of the protein and can be used to optimise the protein construct and the buffer conditions of the sample. Indeed, the quality of the ^15^N–^1^H HSQC depends on the folding and stability of the protein, that in turn is dependent on various parameters such as protein concentration, type of buffer, salt concentration, pH and temperature.

Various types of buffer are suitable for NMR studies and some parameters must be taken into consideration when optimizing buffer conditions. Ideally, the buffer used should not be protonated. Indeed, the concentration of buffer is generally higher than that of the protein and, if protonated, the buffer NMR signals will interfere with the protein signals. The most commonly used NMR buffer is sodium phosphate at a concentration ranging between 10 and 50 mM. Alternatively, other buffers such as Tris–HCl or HEPES can be used at similar concentrations. As these buffers contain protons, it is preferable to purchase them in a deuterated form. NMR sensitivity is inversely correlated with the ionic strength of the buffer. Typically, the ionic strength should be minimal and not exceed 150 or 200 mM sodium or potassium chloride. If protein solubility requires high ionic strength, a high concentration of salt can efficiently be replaced by low-conductivity salts such as 50 mM l-Arginine and l-Glutamate [33]. Additionally, the pH of the buffer must be neutral or slightly acidic (typical range between 5 and 7). Amide protons exchange with the solvent and high pH increases this exchange rate leading to a loss of amide signals in NMR experiments such as ^15^N–^1^H HSQC. Below pH 5, the acidic condition might induce protein unfolding and aggregation. It should also be noted that the pH should be different from the isoelectric point (pI) of the protein or protein domain by at least 0.5 to avoid problems of solubility. Finally, compounds that are protonated or that increase the viscosity of the buffer, such as glycerol, must be avoided.

**Fig. 2:**
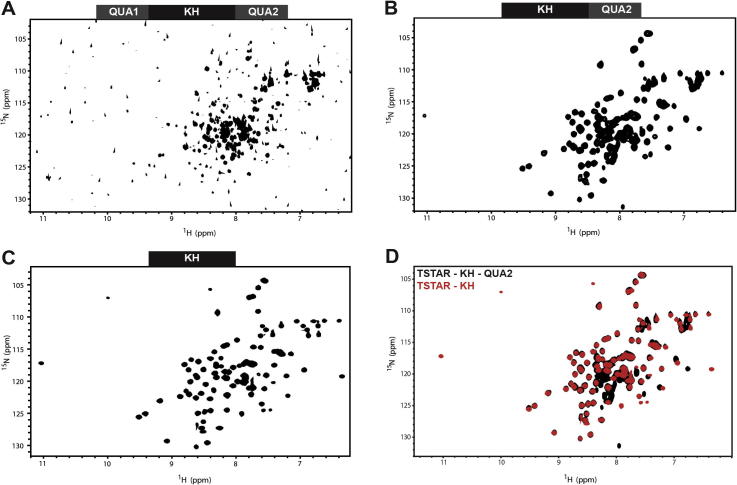
Condition optimisation for NMR studies of T-STAR constructs. ^15^N–^1^H HSQC spectra of T-STAR constructs. (A) STAR domain in 20 mM sodium phosphate pH 6.5, 50 mM NaCl, 30 °C. (B) KH-QUA2 in 20 mM sodium phosphate pH 6.5, 50 mM NaCl, 30 °C. (C) KH in 10 mM Tris–HCl pH 6.5, 50 mM NaCl, 30 °C. (D) Overlay of the ^15^N–^1^H HSQC spectra of T-STAR KH-QUA2 (black) and KH (red).

**Fig. 3:**
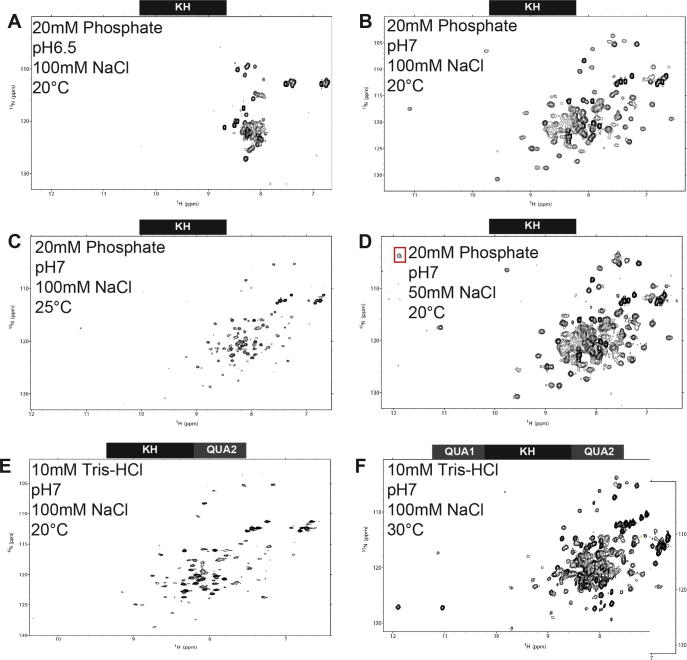
Condition optimisation for NMR studies of Sam68 constructs. ^15^N–^1^H HSQC spectra of Sam68 constructs. (A) KH domain in 20 mM sodium phosphate pH 6.5, 100 mM NaCl, 20 °C. (B) KH in 20 mM sodium phosphate pH 7.0, 100 mM NaCl, 20 °C. (C) KH in 20 mM sodium phosphate pH 7, 100 mM NaCl, 25 °C. (D) KH in 20 mM sodium phosphate pH 7.0, 50 mM NaCl, 20 °C. (E) KH-QUA2 in 10 mM Tris–HCl pH 7.0, 100 mM NaCl, 20 °C. (F) STAR domain in 10 mM Tris–HCl pH 7.0, 100 mM NaCl, 30 °C.

The success of the structure determination depends on the sample conditions, and thus a screen of different conditions can be designed at an early stage of the NMR study to improve the spectra quality. Typically, in our laboratory, as in other laboratories, we initially measure a ^15^N–^1^H HSQC in standard buffer conditions (20 mM sodium phosphate pH 7, 50 mM sodium chloride, 20 °C) and estimate the quality of the spectrum. A screen of conditions is then applied where the pH is varied from 5.5 to 7.5 and the salt concentration from 0 to 200 mM NaCl. For each condition, a ^15^N–^1^H HSQC is measured at 20, 25 and 30 °C and the quality of the spectrum is estimated based on the number and the line width of the visible crosspeaks (Fig. 2, Fig. 3).

#### 3.1.1 Optimisation of T-STAR constructs

In order to obtain a suitable protein sample for NMR studies, we tested different constructs of T-STAR that are expected to be sufficient for RNA binding: the full STAR domain (amino acids 1–183), and shorter constructs containing the KH (50–160) and the KH-QUA2 (50–183) domains. In all cases, the protein constructs expressed very well in *E. coli*, were soluble and could be purified using Ni-NTA agarose followed by TEV cleavage and gel filtration. These protein constructs remained soluble in various buffers suitable for NMR studies and could be concentrated to a final protein concentration above 200 μM.

We started the project by investigating the full STAR domain of T-STAR and preparing a sample of this protein in a common NMR buffer containing 20 mM sodium phosphate pH7, 50 mM sodium chloride and measuring a ^15^N–^1^H HSQC experiment at 20 °C. Despite the fact that the protein is soluble in this buffer condition, the ^15^N–^1^H HSQC spectrum was poorly defined (Fig. 2A). With this protein construct containing 183 amino acids including 8 prolines, one would expect 174 amide crosspeaks in the spectrum. The number of crosspeaks that could be observed in the amide region of the spectrum was only 107. The spectrum shows that most peaks are located in the center of the spectrum and have a high intensity, which is typical for flexible regions of proteins. This clearly indicates that in these conditions, although the protein construct is soluble, the quality of the spectrum is not suitable for structural analysis. Attempts to optimise the sample conditions by varying the pH, the temperature and the salt concentration did not improve the quality of the ^15^N–^1^H HSQC spectrum (data not shown). We then expressed and purified a truncated version of the STAR domain, the KH-QUA2, which lacks the QUA1 dimerisation domain but is expected to be sufficient for RNA binding. This construct was also soluble at suitable NMR concentrations in various buffer conditions and the quality of the ^15^N–^1^H HSQC spectrum improved dramatically (Fig. 2B). Crosspeaks are well-dispersed in the proton dimension indicating that the KH-QUA2 construct is correctly folded. 101 out of 126 crosspeaks were observed. The central region of the spectrum still contains many intense peaks suggesting that some parts of the protein construct are flexible. This is consistent with previous structural studies on the STAR protein Quaking, showing that the QUA2 region is flexible in solution [34]. We thus tested another shorter protein construct of T-STAR, the isolated KH domain. As for the other constructs, the KH domain of T-STAR expressed very well in *E. coli* and remained soluble at concentrations above 200 μM. The ^15^N–^1^H HSQC spectrum of the KH domain was of excellent quality. Condition optimisations for this domain were performed and it appeared that the NMR spectra of this domain remained suitable for NMR studies under various buffer conditions, pH, salt concentration and temperature ranges. From our initial screen, we defined the optimal conditions as 20 mM sodium phosphate pH 6.3, 50 mM NaCl, 30 °C. A final optimisation of these conditions was performed replacing the sodium phosphate by TRIS–HCl or HEPES. We observed that changing the buffer to 10 mM TRIS–HCl pH 7 improved the stability of the sample and the quality of the spectrum, although the buffering capacity of TRIS–HCl is not effective at this pH. These conditions were subsequently used for our NMR studies (Fig. 2C). In these conditions, the ^15^N–^1^H HSQC spectrum of T-STAR KH displayed 100 crosspeaks out of the 103 expected. Furthermore, an overlay of the ^15^N–^1^H HSQC spectra of T-STAR KH and KH-QUA2 shows that the fold of the KH domain is similar in both constructs (overlap of crosspeaks) and that the QUA2 region is flexible since most additional crosspeaks of KH–QUA2 are located in the center of the spectrum and more intense than the crosspeaks corresponding to the KH domain (Fig. 2D).

#### 3.1.2 Optimisation of Sam68 constructs

Sam68 and T-STAR are highly homologous proteins. The main difference between these proteins is the presence of a 100 amino acid N-terminal region of Sam68 that is not present in T-STAR (Fig. 1A). Considering the STAR domain of both proteins, sequence alignment indicates that 69% of the amino acids are identical and 16% display a strong similarity (Fig. 1B). When considering the KH domain only, the identity increases to 77% with a strong similarity of 14%. We therefore anticipated that the KH domain of Sam68 would behave similarly to the KH domain of T-STAR in solution and initiated an NMR study of the Sam68 KH construct using the optimal conditions defined for T-STAR (10 mM Tris pH 6.5, 50 mM NaCl, 30 °C). Sam68 KH expressed well and was soluble in *E. coli*, although with a lower yield than T-STAR KH. The affinity chromatography purification procedure was the same as for T-STAR. Dialysing the protein in the T-STAR NMR buffer, however, resulted in a large amount of precipitation and we could not recover soluble forms of Sam68 KH. Changing the buffer from 10 mM TRIS–HCl to 20 mM sodium phosphate and increasing the salt concentration of the dialysis and gel filtration buffers to 100 mM NaCl allowed us to maintain the solubility of the protein. We could obtain a sample of ^15^N labelled Sam68 KH at approximately 0.2 mM that was sufficient for measuring a ^15^N–^1^H HSQC experiment at 20 °C (Fig. 3A). All amide crosspeaks were very intense and located in the central region of the spectrum, indicating that in these conditions only the flexible regions of Sam68 KH were visible. We performed a screen of conditions as described above. In summary, increasing the pH to 7.0 led to the appearance of well-dispersed crosspeaks at 20 °C (Fig. 3B). Increasing the temperature to 25 °C resulted in sample precipitation (Fig. 3C). Finally, decreasing the NaCl concentration from 100 to 50 mM, improved the signal to noise ratio of the spectrum (Fig. 3D). These conditions could be used to investigate the binding of RNA to Sam68 KH domain (see Section 3.2.2.), although the protein could not be kept in its stable folded state for a long period of time. For this reason, we recently tested the expression and solubility of alternative constructs of Sam68. Initially, the KH–QUA2 domain was expressed and purified using the same protocol as for the KH domain. In the same sample conditions as Sam68 KH, we were unable to concentrate this construct adequately and the protein was unstable, even at 20 °C (Fig. 3E). The full STAR domain of Sam68 was then expressed and yielded larger amounts of protein than either the KH or KH–QUA2 constructs. We were able to concentrate this sample up to ∼500 μM and it remained stable at 30 °C for a long period of time (several weeks at room temperature), making it highly suitable for NMR analysis. The ^15^N–^1^H HSQC spectrum shows that, in contrast to T-STAR STAR, the STAR domain of Sam68 is well folded and we observed 172 crosspeaks out of 174 expected (Fig. 3F). In addition, the spectrum of Sam68 STAR overlays well with the spectrum of isolated KH domain and of isolated QUA1 [21], suggesting that the QUA1 dimerisation domain and the KH domain of Sam68 are properly folded in our STAR construct.

### 3.2 Defining the optimal ssRNA sequence for NMR studies

As described in the previous section, the ^15^N–^1^H HSQC spectrum can be considered the fingerprint of the protein. Since the frequency of each nucleus depends on its chemical environment, NMR can be used to investigate the binding of partner molecules to a protein. The NMR chemical shift perturbation assay consists of adding increasing amounts of unlabeled partner to a ^15^N labeled protein and measuring ^15^N–^1^H HSQC experiments for various partner-protein molar ratios [35], [36]. If binding occurs, the amino acids at the interface with the partner will experience a different chemical environment and therefore their chemical shift will be different. This experiment provides precise information on the complex formation, such as an estimation of the dissociation constant, the stoichiometry of the complex and the amino acids involved in the interaction. In solution, the protein and the RNA are in equilibrium between their free and bound states and this equilibrium depends on the dissociation constant of the complex. During the NMR experiment, depending on the exchange rate of the complex formation, three different events can occur. In the slow exchange regime, the progressive addition of RNA leads to the presence of two crosspeaks for one perturbed N–H, one corresponding to the free and one to the bound form of the protein. The intensity of each crosspeak is directly proportional to the protein:RNA molar ratio. This exchange regime is reported for protein–RNA complexes with high affinities (dissociation constant below 200 nM). In the fast exchange regime, only one signal corresponding to an average of the free and bound state of the protein is visible. The addition of increasing amounts of RNA will gradually shift the signal from the free state towards the bound state of the protein. This exchange regime is generally reported for protein–RNA complexes with relatively low affinities (dissociation constant higher than 20 μM). Finally, in the intermediate exchange regime, crosspeaks tend to disappear upon addition of RNA due to line broadening and reappear when the stoichiometry of the complex is reached. In many cases however, crosspeaks do not reappear, even in the presence of excess RNA. In that case, optimisation of the conditions (buffer, salt concentration, temperature) should be performed to obtain a suitable NMR spectrum of the protein–RNA complex. The intermediate exchange regime is reported for protein–RNA complexes with dissociation constants between 400 and 2 μM. NMR chemical shift perturbation experiment is very powerful and allows screening of different RNA sequences at an early stage of the structural work, permitting the identification of the optimal RNA sequence for structural investigation of protein–RNA complexes.

Chemical shift perturbation experiments performed using ^15^N–^1^H HSQC experiments, as detailed above, only provide information on the quality of the protein NMR signals. In addition, it is important to investigate the quality of the RNA signals, since the structure determination of the complex will rely on NMR derived restraints from both the protein and the RNA. As short single-stranded RNAs are mainly obtained by chemical synthesis, they can not be easily labeled isotopically. Observing solely the RNA resonances in the protein–RNA complex can therefore only be achieved by labeling proteins with ^15^N and ^13^C and using specific NMR experiments that cancel protein signals (reviewed in [37]). Since certain RNA chemical shifts are distinct from the protein ones, it is still possible to evaluate the quality of the RNA spectra using proton NMR experiments such as 2D DQF-COSY (Double Quantum Filtered Correlation Spectroscopy), 2D TOCSY (Total Correlation Spectroscopy) and 2D NOESY (Nuclear Overhauser Effect Spectroscopy) experiments (for more details, see [36]) without the need for producing ^15^N/^13^C-labeled protein samples. Homonuclear DQF-COSY and TOCSY are through-bond NMR experiments. Crosspeaks are observed between protons connected by two or three covalent bonds. For example, in RNA pyrimidines, the base contains two protons, H5 and H6, connected by three bonds through carbon atoms. Homonuclear NOESY is a through-space NMR experiment. Crosspeaks are observed between protons that are close in space (typically less than 5 Å). This experiment is crucial in NMR structure determination for obtaining inter-proton (intra-protein, intra-RNA and intermolecular) distance restraints.

In order to design a pool of RNA targets for NMR screening, prior knowledge of the protein–RNA specificity is highly desirable. Several biochemical methods allow the identification of specific RNA sequences bound by RBPs, including footprinting, Systematic evolution of ligands by exponential enrichment (SELEX) or Cross-linking immunoprecipitation (CLIP) techniques. Footprinting experiments have been used for decades, using enzymes or chemicals that specifically cleave RNA molecules at certain positions, allowing the investigation of RNA structures and the identification of RNA sequences specifically bound by RBPs [38]. SELEX is an *in vitro* method consisting of a series of selection cycles of interacting RNA from a randomised oligonucleotide library. This generally allows for the identification of a consensus RNA sequence bound by the protein of interest [39]. CLIP experiments make use of the fact that UV irradiation of sample material, such as a cell lysate, causes covalent bond formation between RNA and proteins [40], [41]. This technique allows the identification of natural RNA targets for the protein of interest and a consensus RNA sequence can be derived. Alternatively, when no specific sequence is known to bind an RBP, an NMR based method, called scaffold-independent analysis (SIA), has been developed using short synthetic randomised RNA sequences that are tested for binding to an RBP or RBD by NMR ^15^N–^1^H HSQC [42].

Each of these techniques provides useful preliminary information to define a pool of RNA sequences to screen for protein–ssRNA complex structure determination. It should be noted that consensus sequences derived from CLIP, SELEX or SIA are often degenerate and differ from natural sequences bound by RBDs. Nonetheless, the optimal RNA sequence for structure determination is not necessarily found naturally, nor has the highest affinity for the protein. This is due to the fact that a precise structure determination of a protein–RNA complex requires a single and stable conformation of the complex. For example, natural and/or high affinity RNA sequences often contain multiple, similar, and juxtaposed binding sites and are not suitable for structural work because the protein can bind these sequences in multiple registers leading to an inhomogeneity of the sample and a loss of NMR signal. It is therefore crucial to identify the optimal RNA sequence that has reasonably high affinity to obtain a stable complex, specificity to obtain a homogeneous complex, and is still similar to natural sequences to derive biologically relevant structural information.

**Fig. 4:**
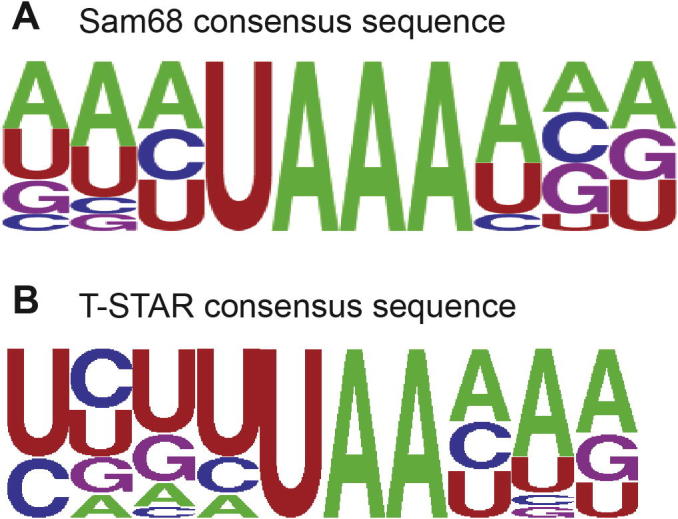
Consensus RNA sequences derived from SELEX experiments. (A) Sam68 derived consensus RNA sequence. (B) T-STAR derived consensus RNA sequence. Figures were generated using WEBLOGO [57].

Structure analysis of various KH domains in complex with DNA or RNA showed that the classical nucleotide binding pocket of KH domains accommodates 4 nucleotides and structures of these complexes were solved with DNAs or RNAs varying from 4 to 12 nucleotides in length [25]. In the case of Sam68, SELEX experiments defined three consensus RNA motifs with different binding affinities, UAAA having the highest affinity, followed by UUUA and AAAA [26], [27]. Accordingly, these motifs have been identified in numerous pre-mRNAs bound by Sam68 [43], [44], [45], [46], [47], [48]. However, other RNA sequences have been identified in other pre-mRNAs, such as AAAUU [49], [50]. Interestingly, it has been recently reported that Sam68 bound a UAAUAAA motif present in the Neurexin pre-mRNA but not a truncated RNA containing only the UAAA motif [51]. Finally, Sam68 was also shown to bind poly(U) RNA sequences [52], [53]. In the case of T-STAR, SELEX experiments identified A/U-rich sequences similar to the one bound by Sam68 [27]. Recently, a novel method, RNAcompete, defined the core binding site of T-STAR as UAA [54]. Similar AU-rich motifs have also been identified by CLIP experiments (S. Grellscheid, D. Elliot, personal communication). Finally, NMR-based SIA experiments with T-STAR KH suggested a preference for A-rich RNA sequences (K. Collin, A. Ramos, personal communication). The biological role of T-STAR is still unclear, and only one pre-mRNA target has been identified to date with a T-STAR binding site defined as 4 × (UUAA) [19]. Interestingly, Sam68 and T-STAR share 77% identity in their KH domains and correspondingly both proteins bind A/U rich RNA sequences. Yet, a comparison of the SELEX outputs suggest that these two proteins could specifically bind slightly different RNA sequences which could explain the fact that these proteins are not biologically equivalent (Fig. 4) [19]. Indeed, Sam68 seems to favour a UAAA motif surrounded preferentially by A (Fig. 4A), while T-STAR favours a UAA motif preferentially preceded by U and followed by A (Fig. 4B). Based on these consensus sequences, we have designed a series of 6mer A/U-rich RNAs (Table 1). For instance, sequences AAAUAA and AAUAAA resemble the Sam68 consensus sequence; UUUAAA resembles the T-STAR consensus sequences, while sequences like UAAAAA resemble both Sam68 and T-STAR consensus sequences. In addition, other sequences were derived based on pre-mRNA target sites such as AAAUUU and UAAAUU. Finally, we designed derivatives of these sequences, as well as 6mer polyA and polyU. Series of longer and shorter RNAs were also designed to reflect natural targets of Sam68 (UAAUAAAUU) or T-STAR (UAAUUAAA and AUUAAUUA) and to investigate whether the length of the optimal RNA sequence could improve the structural quality of the protein–RNA complex (Table 2).

**Table 1:** List of 6mer RNAs used to study the T-STAR–RNA and Sam68–RNA complexes.

AAAUAA	AAUAAA	UUUAAA	UAAAAA	AAAUUU
UAAAUU	UAAAUA	UAAAAU	AAAUAU	AAUAUU
AUUAAA	AAUUUU	AUUUUU	AAAAAA	UUUUUU

**Table 2:** List of RNA with various lengths used to study the T-STAR–RNA and Sam68–RNA complexes.

UAAUAAAUU	UAAUUAAU	AUUAAUUA	UUUAAAUAA	AAAAAAUAA
UAAAUAAUU	UAAAAAUUUU	UAAAAUUUUU	UAAAUUUUUU	UAAAUAUUUU
AAAU	AAUA	AUAA	AAAUA	AAUAA

#### 3.2.1 Defining the optimal ssRNA sequence bound by T-STAR KH

As mentioned in Section 3.1.1, T-STAR KH and T-STAR KH–QUA2 constructs are highly soluble and stable, and the ^15^N–^1^H HSQC spectra of these domains were of excellent quality. In contrast, T-STAR STAR construct resulted in poor NMR spectra. As it has been shown for other STAR proteins that the KH–QUA2 region is sufficient for RNA binding [20] and that in the case of Sam68 (and by homology of T-STAR), the QUA2 region might not be involved in RNA binding [26], [28], we tested the RNA binding ability of the constructs KH–QUA2 and KH. NMR chemical shift perturbation experiments were performed by measuring a ^15^N–^1^H HSQC experiment of a 0.2 mM sample of the free protein as reference. RNA was then gradually added to the protein sample at different molar ratios (protein:RNA ratio of 1:0.5 and 1:1). In all cases, the pH of the RNA stock solution was adjusted to correspond to the pH of the protein solution and RNAs were prepared at high concentration (up to 4 mM) to restrict the issue of RNA to be added to the protein and avoid a dilution of the protein that could affect the chemical shifts.

**Fig. 5:**
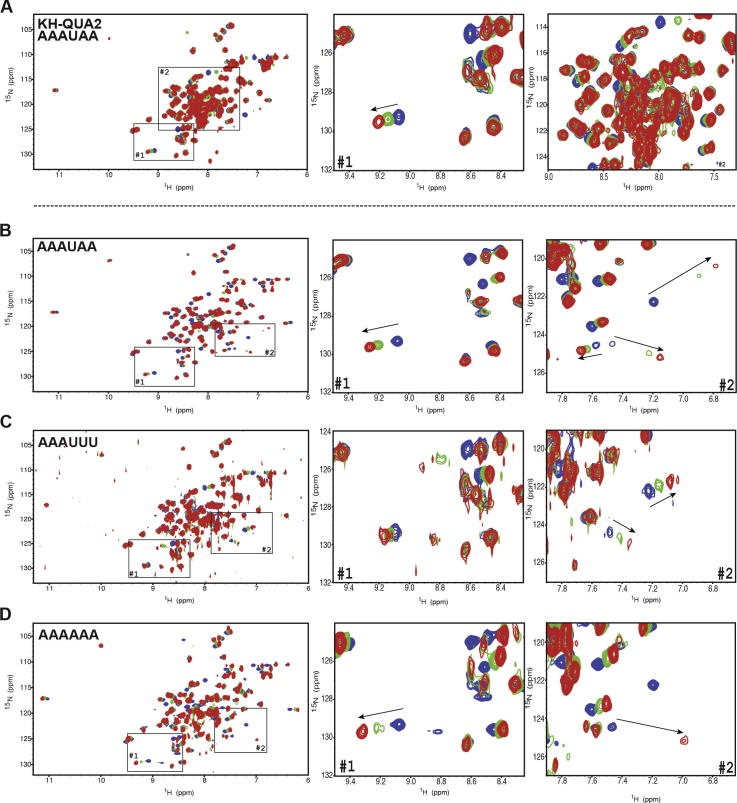
Effect of RNA sequences on T-STAR–RNA complex formation. Chemical shift perturbation experiments of (A) T-STAR KH–QUA2 with AAAUAA, and T-STAR KH with (B) AAAUAA, (C) AAAUUU, and (D) AAAAAA. In all cases, an overlay of ^15^N–^1^H HSQC spectra is displayed for the free protein (blue), a protein:RNA molar ratio of 1:0.5 (green) and a protein:RNA molar ratio of 1:1 (red).

We initially tested the binding of T-STAR KH–QUA2 with some of our 6mer RNAs. With all tested RNA sequences, we observed changes of the protein ^15^N–^1^H HSQC spectrum, some peaks disappearing and others changing position, clearly indicating that these RNA sequences are able to bind T-STAR KH–QUA2 (Fig. 5A). A careful analysis of the chemical shift perturbations showed that all the peaks affected by the RNA addition corresponded to amino acids of the KH domain, while peaks of the QUA2 in the central region of the spectrum were not affected. This suggested that the KH domain of T-STAR could be sufficient for RNA binding. We therefore performed the same experiments with the T-STAR KH construct and indeed observed that the KH domain is sufficient for RNA binding and the chemical shift perturbation observed on the KH construct were similar to those observed on the KH–QUA2 construct. Further screening of RNA sequences was therefore performed on the KH construct of T-STAR.

All the RNA sequences tested showed a clear binding to the KH domain. Typical examples are displayed in Fig. 5B–D. In all cases, the same protein crosspeaks were affected, indicating that, whatever the A/U-rich RNA sequence, the same amino acids are involved in binding. However, the effect of RNA addition on the crosspeaks varied significantly with different RNA sequences (Fig. 5 B-D). For example, the AAAUAA RNA shows a clear fast exchange regime, with crosspeaks gradually shifting from their free to their bound position as a function of the protein: RNA molar ratio. This allows us to follow all the chemical shift perturbation and obtain a complete spectrum of the bound form of the protein (Fig. 5B). Other RNA sequences such as AAAUUU induce chemical shift perturbation in the protein crosspeaks but the intensity of the shift is weaker indicating that these RNAs have a lower affinity for the protein than AAAUAA (Fig. 5C). Other RNAs such as polyA induce perturbations similar to AAAUAA, but some peaks disappeared indicating a fast to intermediate exchange regime (Fig. 5D). Unfortunately, while this implies that these RNA sequences have a higher affinity for the protein, the peaks that disappear do not reappear in the spectrum even in excess of RNA, which is not optimum for acquisition of sufficient data for the structure determination of the protein–RNA complex. Taken together, the analysis of 6-mer RNA sequences showed that they all bound the T-STAR KH protein construct, but with different affinities, leading to different intermediate or fast exchange regimes. Our study showed that the RNA sequence AAAUAA was the optimal one because it induced the largest chemical shift perturbations of the protein crosspeaks and all crosspeaks were visible in the bound state.

**Fig. 6:**
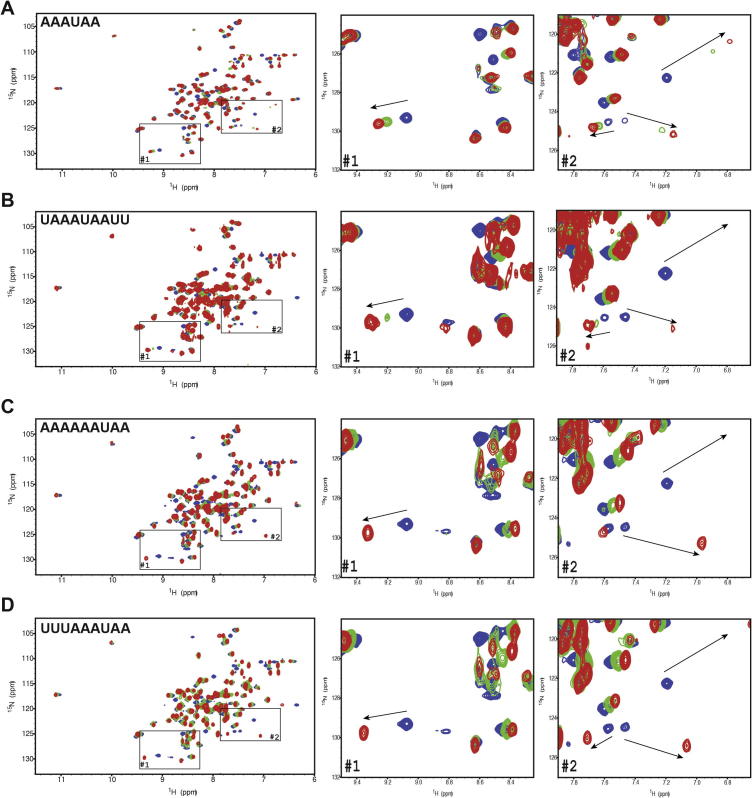
Effect of RNA size on T-STAR–RNA complex formation. Chemical shift perturbation experiments of (A) T-STAR KH with AAAUAA (similar to Fig. 3B), (B) AAAAAAUAA, and (C) UUUAAAUAA. In all case, an overlay of ^15^N–^1^H HSQC spectra is displayed for the free protein (blue), a protein:RNA molar ratio of 1:0.5 (green) and a protein:RNA molar ratio of 1:1 (red).

We then investigated whether the length of the RNA sequence could influence the quality of the NMR spectra. Various derivatives of the AAAUAA sequence were synthesised (Table 2). This included shorter RNA sequences (5mers and 4mers) as well as longer sequences with extension in 5′, 3′ or both. Shorter versions of the RNA sequence were still sufficient for binding the protein but the chemical shift perturbations were smaller than with the 6mer sequence suggesting a lower affinity (data not shown). We then tested longer RNA sequences (9mers) with polyA or polyU extensions in the 5′ or 3′ of the AAAUAA central part. With these RNAs, the chemical shift perturbations have the same effect as the 6mer sequence on the KH domain. They affect the same area of the spectrum but instead of a clear chemical shift perturbation, crosspeaks disappeared and reappeared indicating an intermediate exchange regime and meaning a higher affinity of the protein for these RNAs compared to AAAUAA. However, not all crosspeaks of the protein reappeared when fully bound and these longer RNAs were therefore not suitable for structure determination (Fig. 6). In conclusion, we optimised both the composition and the length of the RNA sequences bound to T-STAR KH and concluded that the optimal sequence for structure determination was AAAUAA. Interestingly, this sequence could not be derived from the T-STAR specific RNA consensus sequence, but resemble the Sam68 consensus. Nevertheless, this sequence still contains the UAA core consensus sequence for T-STAR.

**Fig. 7:**
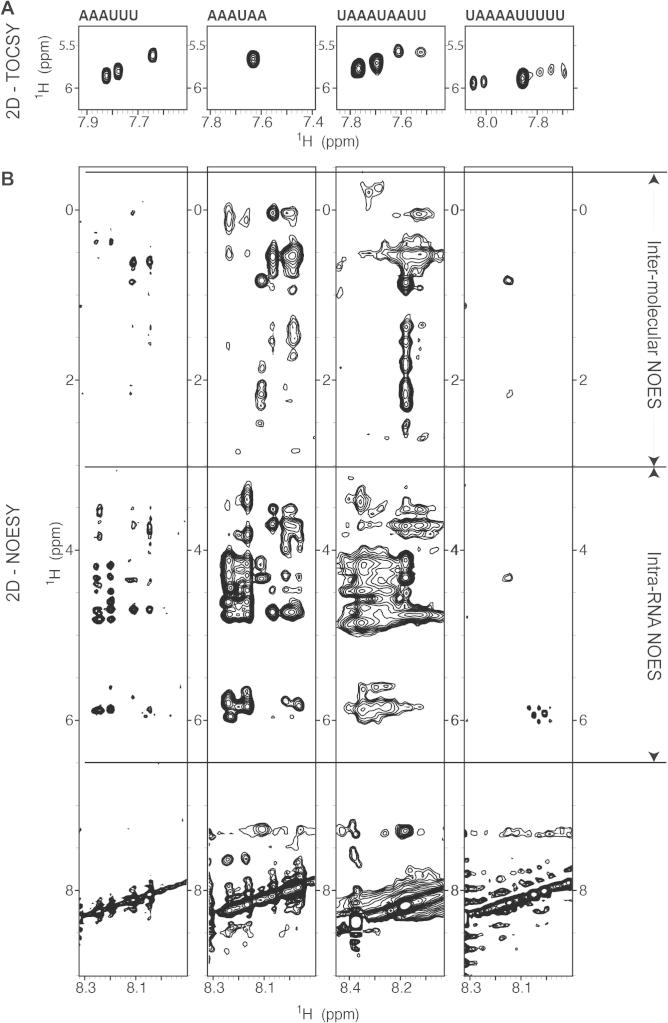
Analyzing the NMR resonances of various RNAs in complex with T-STAR KH. (A) TOCSY and (B) NOESY spectra of different RNA sequences in complex with T-STAR KH measured in D_2_O. The displayed section of the TOCSY spectra shows to the H5/H6 region of RNA pyrimidines and the section of the NOESY spectra shows the NOES between the RNA bases and either the RNA sugars (intra-RNA NOES) or the protein (intermolecular NOES).

TOCSY and NOESY NMR experiments have also been used to investigate the NMR signal quality of the different RNA sequences in complex with T-STAR KH. As most RNA base protons are non-labile and have chemical shift values overlapping with the amide protein protons and with water, these experiments were recorded in 100% D_2_O (see Section 2). In these conditions, the amide protons of the protein exchange with deuterium and the RNA crosspeaks can easily be analyzed. TOCSY spectra were used to identify the crosspeaks of the uridine H5–H6 bases. As expected, the TOCSY spectra of the AAAUAA and AAAUUU RNAs in complex with T-STAR KH displayed one and three crosspeaks, respectively, indicating that the uridine bases experience a single chemical environment when bound to the protein (Fig. 7A). In contrast, the TOCSY spectra of the longer sequences UAAAUAAUU and UAAAAUUUUU displayed two and one intense crosspeaks, instead of the four and six expected (Fig. 7A). This indicates that chemical exchange of these protons occur during binding and could be due to the RNA binding the protein in different registers. NOESY spectra provide useful information on the quality of the complex for NMR studies. When measured in 100% D_2_O, the resonances in the 8 ppm frequency region correspond mainly to the RNA bases (in our case, adenine H8 and H2 and uridine H6). Crosspeaks from this region of the spectrum to the RNA sugar region (3–6.5 ppm) arise from RNA base protons in close proximity to RNA sugar protons (intra-RNA NOES) while crosspeaks to other regions of the spectrum (0–3 ppm) arise from RNA base protons in close proximity to protein protons (intermolecular NOES). As shown in Fig. 7B, the NOESY spectrum of AAAUAA in complex with T-STAR KH displays many NOE crosspeaks in the intra-RNA region, suggesting that the RNA adopts a well-defined conformation and is not disordered. Many NOES can also be observed in the intermolecular region, suggesting that the protein–RNA complex adopts a well-defined orientation and that intermolecular distances can be extracted, which are crucial for the structure determination of a protein–RNA complex by NMR. In contrast, the NOESY spectra of the other RNAs in complex with T-STAR KH displayed no or few intra-RNA and intermolecular NOES indicating that these RNA sequences are not suitable for structure determination of the protein–RNA complex. These NMR experiments confirmed our previous conclusion that the RNA sequence AAAUAA is the optimal sequence for the NMR structure determination of the T-STAR KH-RNA complex.

#### 3.2.2 Defining the optimal ssRNA sequence bound by Sam68 KH

**Fig. 8:**
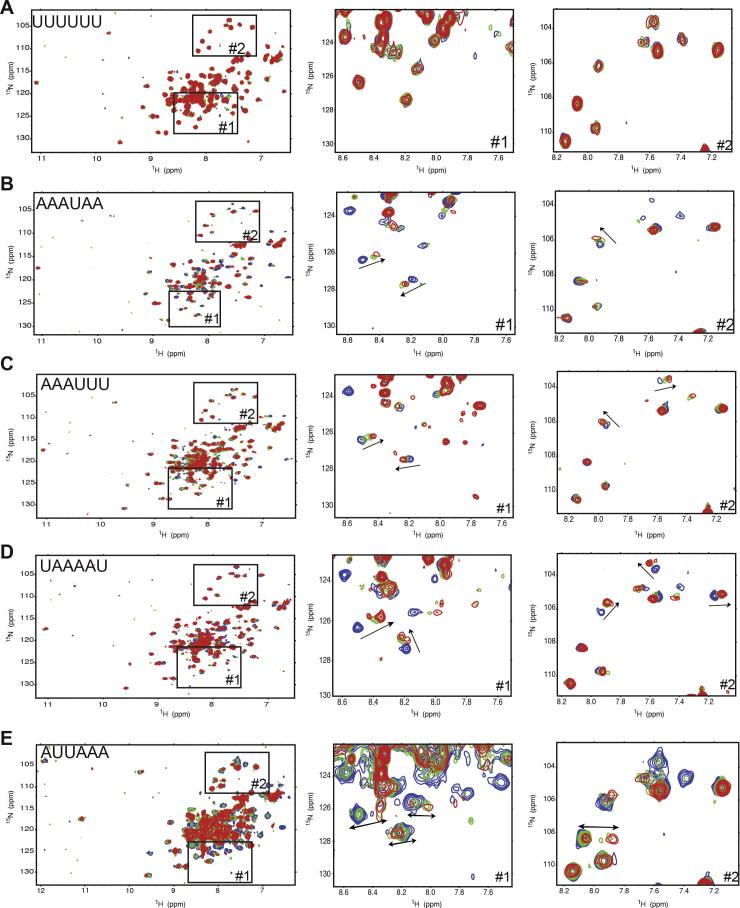
Effect of RNA sequences on Sam68-RNA complex formation. Chemical shift perturbation experiments of Sam68 KH domain with (A) UUUUUU, (B) AAAUAA, (C) AAAUUU, (D) UAAAAU and (E) AUUAAA. In all case, an overlay of ^15^N–^1^H HSQC spectra is displayed of the free protein (blue), a protein:RNA molar ratio of 1:0.5 (green) and a protein:RNA molar ratio of 1:1 (red).

Given the high sequence homology between Sam68 and T-STAR KH, we performed chemical shift perturbation experiments on the KH domain of Sam68 with various AU-rich 6mer RNAs. The quality of the spectra was not as good as that of T-STAR KH, and the sample was not as stable (see Section 3.1.2). It was however sufficient to identify changes in the protein spectrum upon addition of increasing amounts of RNA. This suggested that, as for T-STAR, the KH domain of Sam68 is sufficient for RNA binding. We tested different 6mer RNAs designed according to SELEX and published biological data (Table 1). Interestingly, while Sam68 has previously been shown to bind poly(U) RNAs [52], [53], the addition of UUUUUU RNA to Sam68 KH did not affect the NMR spectrum, indicating that, in our conditions, Sam68 KH does not bind poly(U) (Fig. 8A). All other tested RNAs affected the ^15^N–^1^H HSQC spectrum of Sam68 KH, indicating complex formation. Furthermore, the same peaks of Sam68 KH were affected by the addition of RNA, suggesting that the same residues are involved in binding. Different RNA sequences led to a combination of intermediate and fast exchange regimes, with many peaks disappearing and others shifting upon RNA addition. Surprisingly, crosspeaks in fast exchange shifted in different directions depending on the RNA sequence used (Fig. 8B–D), indicating that the chemical environment of these amino acids is different when bound to different RNA sequences. This suggests that although the same amino acids are affected by the various RNAs, the KH domain binds these RNAs in a slightly different way. An interesting RNA sequence is AUUAAA. The chemical shift perturbation experiment with this RNA was in the slow exchange regime indicating a strong affinity for the protein (Fig. 8E). In this case, most crosspeaks corresponding to the bound form of the protein were visible, making it a suitable candidate for further structural studies. However, since the sample was not stable, the quality of the ^15^N–^1^H HSQC spectrum remained poor and we could not measure additional NMR experiments such as NOESY.

Recently, we have produced samples of the STAR domain that are stable (Fig. 3F). These new samples are suitable for NMR structural studies and we will therefore investigate the binding of the different A/U-rich RNA sequences to the STAR domain of Sam68.

### 3.3 Using NMR data to optimise crystallisation conditions

**Fig. 9:**
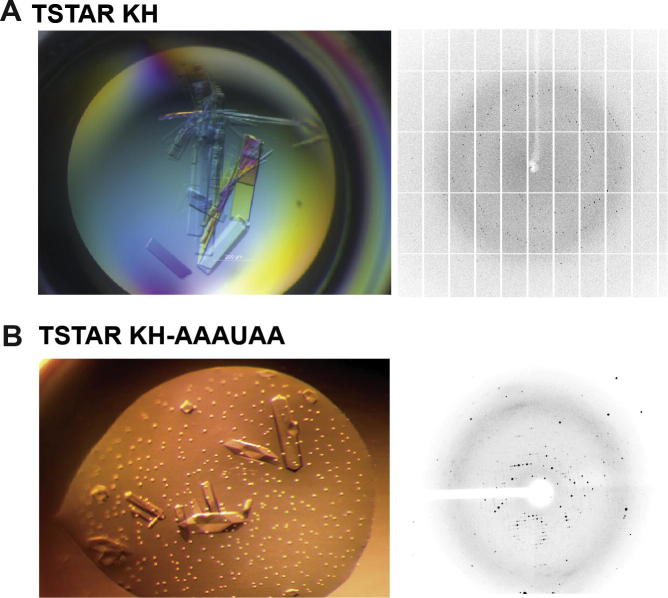
Using NMR screening for X-ray crystallography. Crystals and diffraction pattern of (A) free T-STAR KH and (B) T-STAR KH–AAAUAA complex.

X-ray crystallography is the primary method to determine the molecular structure of various biological molecules. This requires the molecules to aggregate in a well-ordered crystal. The principal factor for crystallisation is the buffer composition that, as for NMR, must be optimised. Because our NMR analysis showed that the KH domain of T-STAR was highly soluble and structured in solution (see Section 3.1.1), we set up crystallisation screens for this domain using six commercially available screens and protein concentrations ranging from 10 to 20 mg ml^−^^1^ in our optimised NMR buffer. We obtained various hits and optimised the conditions in order to obtain protein crystals of sufficient size. Our optimised crystals were rectangular and diffracted to a resolution of 1.6 Å (Fig. 9A). Interestingly, we observed that, in contrast to T-STAR KH, Sam68 KH does not behave well in solution (Section 3.1.2). Accordingly, crystallisation trials of Sam68 KH did not produce any crystal hits suggesting that NMR preliminary experiments on the solubility and stability of proteins (Sections 3.1.1 and 3.1.2) can provide useful information for crystallisation trials of proteins.

Using NMR chemical shift perturbation experiments, we have tested a large number of RNA sequences for binding T-STAR KH (Section 3.2.1) and concluded that the AAAUAA RNA sequence was the most suitable candidate for the structure determination of T-STAR KH in complex with RNA (Section 3.2.1). We therefore initiated a crystallisation trial of T-STAR with various 6-mer RNA sequences. Interestingly, only the complex of T-STAR with the AAAUAA RNA crystallised. In this case, crystals were hexagonal and diffracted to a resolution of 2.0 Å (Fig. 9B). Interestingly, the crystallogenesis condition and the space group are different than from the free KH suggesting that these crystals contain both protein and RNA. Furthermore, these data suggest that NMR chemical shift perturbation experiments of protein–RNA complexes can be used as a screening method to optimise the crystallisation procedure of such complexes.

## 4 Concluding remarks

Over the past few years, there has been an increasing interest in RNA biology and RNA binding proteins. Structural studies of protein–RNA complexes are therefore needed if we want to understand how proteins recognise specifically their RNA targets and to derive a general code for RNA recognition [55], [56]. The intrinsic properties of such complexes, however, make them difficult to study structurally. In this article, we have shown how NMR can be used at an early stage of structural studies to first identify which protein constructs are suitable and, second to screen many RNA sequences in order to identify the optimal protein–RNA complex for structure determination.
